# Nitrate of Silver to Prevent Ophthalmia Neonatorum

**Published:** 1882-11

**Authors:** 


### NITRATE OF SILVER TO PREVENT OPHTHALMIA NEONATORUM.

Crede's procedure* for procedure for preventing inflammation of the eyes of the infants, was used during the year 1881, on 361 children in the Obstetrical School in Stuttgart, with the best possible results, for not one case of ophthalmia occurred during the year.—*Bayer, Arch. f. Gyn. xix, p.* 258.

* Crede's method consists in dropping into both eyes of the infant a one per cent. solution of argent. nitratum.

